# Association between personal exposure to household air pollution and gestational blood pressure among women using solid cooking fuels in rural Tamil Nadu, India

**DOI:** 10.1016/j.envres.2022.112756

**Published:** 2022-05-15

**Authors:** Wenlu Ye, Gurusamy Thangavel, Ajay Pillarisetti, Kyle Steenland, Jennifer L. Peel, Kalpana Balakrishnan, Shirin Jabbarzadeh, William Checkley, Thomas Clasen, the HAPIN Investigators

**Affiliations:** a Gangarosa Department of Environmental Health, Rollins School of Public Health, Emory University, Atlanta, GA, USA; b Department of Environmental Health Engineering, Sri Ramachandra Institute of Higher Education and Research, Chennai, Tamil Nadu, India; c Department of Biostatistics and Bioinformatics, Rollins School of Public Health, Emory University, Atlanta, Georgia, USA; d Dept of Environmental and Radiological Health Sciences, Colorado State University, Fort Collins, CO, USA; e Division of Pulmonary and Critical Care, School of Medicine, Johns Hopkins University, Baltimore, MD, USA; f Center for Global Non-Communicable Disease Research and Training, School of Medicine, Johns Hopkins University, Baltimore, MD, USA; g Environmental Health Sciences, School of Public Health, University of California, Berkeley, California, USA

**Keywords:** Household air pollution, Solid fuels, Pregnant women, Blood pressure

## Abstract

**Background:**

The Household Air Pollution Intervention Network (HAPIN) trial is an ongoing multi-center randomized controlled trial assessing the impact of a liquified petroleum gas (LPG) cookstove and fuel intervention on health. Given the potential impacts of household air pollution (HAP) exposure from burning solid fuels on cardiovascular health during pregnancy, we sought to determine whether baseline exposures to particulate matter with an aerodynamic diameter ≤2.5 μm (PM_2.5_), black carbon (BC) and carbon monoxide (CO) were associated with blood pressure among 799 pregnant women in Tamil Nadu, India, one of the HAPIN trial centers.

**Methods:**

Multivariable linear regression models were used to examine the association between 24-h personal exposure to PM_2.5_/BC/CO and systolic and diastolic blood pressure, controlling for maternal age, body mass index (BMI), mother's education, household wealth, gestational age, and season. At the time of measurement, women were between 9- and 20-weeks of gestation.

**Results:**

We found that systolic blood pressure (SBP) and diastolic blood pressure (DBP) were higher in pregnant women exposed to higher levels of HAP, though only the result for CO and DBP reached conventional statistical significance (p < 0.05). We observed a positive association between CO and DBP among the entire study cohort: a 1-log μg/m^3^ increase in CO exposure was associated with 0.36 mmHg higher DBP (95% confidence interval [CI]: 0.02 to 0.70). The effect was stronger in pregnant women with higher CO exposures (in the 3rd [≥ 0.9 and < 2.1 ppm] and 4th quartiles [≥ 2.1 and ≤ 46.9 ppm]). We also found that pregnant women with PM_2.5_ exposures in the highest quartile (≥ 129.9 and ≤ 2100 μg/m^3^) had a borderline significant association (p = 0.054) with DBP compared to those who had PM_2.5_ exposures in the lowest quartile (≥ 9.4 and < 47.7 μg/m^3^). No evidence of association was observed for BC exposure and blood pressure.

**Conclusion:**

This study contributes to limited evidence regarding the relationship between HAP exposure and blood pressure among women during pregnancy, a critical window for both mother and child's life-course health. Results from this cross-sectional study suggest that exposures to PM_2.5_ and CO from solid fuel use are associated with higher blood pressure in pregnant women during their first or second trimester.

## Highlights

• Pregnant women using solid fuels are exposed to high household air pollution levels.
• Blood pressure was higher among pregnant women exposed to higher levels of PM and BC.
• Household air pollution exposure may increase blood pressure in pregnant women.

## 1 Introduction

Although the use of solid fuels has steadily fallen in past decades, nearly half of the world's population – about 3.8 billion people – continue to rely on wood, animal dung, charcoal, and agricultural residues for cooking and heating (Health Effects Institute, 2020). In India, the proportion of the population using solid fuels for cooking was 56.3% in 2019 (Pandey et al., 2020). Burning solid fuels in poorly ventilated homes can result in dangerous levels of exposure to harmful household air pollutants, including fine particulate matter with an aerodynamic diameter of less than 2.5 μm (PM_2.5_), black carbon (BC), and carbon monoxide (CO), among others. The most recent Global Burden of Disease (GBD) study reported that household air pollution (HAP) generated from solid fuel use contributed an average of 82.8 μg/m^3^ PM_2.5_ in Indian households (Pandey et al., 2020). In 2019, exposures to HAP are estimated to have accounted for more than six hundred thousand deaths and 6.7% of total disability-adjusted life years (DALYs) in India (Pandey et al., 2020). The main contributor to this disease burden is cardiovascular disease, and high systolic blood pressure (SBP) is the leading risk factor for cardiovascular diseases in India (Pandey et al., 2020).

Elevated blood pressure has been linked to air pollution (particularly PM_2.5_ exposure) through an imbalance of lung autonomic nerve function, systematic oxidative stress, inflammation, and endothelial dysfunction (Brook et al., 2009, 2010, 2011). The association between air pollution exposure and blood pressure is based mainly on findings from ambient air pollution studies (Brook et al., 2009; Liang et al., 2014; Paolo et al., 2015). Comparatively few studies have examined the association between blood pressure among women exposed to HAP, and those that have report inconsistent findings (Alexander et al., 2015; Baumgartner et al., 2011, 2014, 2018, 2011; Clark et al., 2011, 2013, 2019; Dutta et al., 2011; Dutta and Ray, 2014; McCracken John et al., 2007; Norris et al., 2016; Young et al., 2019). Conflicting results might be due to heterogeneity in study location, population, fuel type, exposure patterns, and HAP source and composition.

The potential of HAP exposure to adversely affect blood pressure presents a special concern in pregnant women. Hypertension among Indian women appears to be rising; the National Family Health Survey-4 in India (NFHS-4, 2014–2015) reported a 10% prevalence of hypertension among Indian women, which increased to 22.5% in NFHS-5 (NFHS-5, 2019–2020). A study on preeclampsia estimated the hypertensive disease of pregnancy was 10.3% in South India (Magee et al., 2019) and another large hospital based study conducted in Tamil Nadu estimated the prevalence of 10.4% (Sengodan and Sreeprathi, 2019). Given this background, studying the adverse effect of HAP exposures on blood pressure in pregnant women in India is of importance, as they are the primary cooks in many households and are exposed to dangerous levels of HAP on a daily basis. Even minor shifts in blood pressure associated with HAP exposure during this vulnerable life stage could have important health implications, especially at population scales. Hypertension during pregnancy can be a symptom of an underlying syndrome with substantial maternal and fetal morbidity and mortality (Yoder et al., 2009). A nationally representative Indian survey showed that 7.1% of maternal mortality was associated with hypertensive disorders of pregnancy (Montgomery et al., 2014). Early recognition of blood pressure elevation and hypertensive disorders during pregnancy and its risk factors are crucial to avoid adverse maternal outcomes in late-pregnancy or at delivery.

To the best of our knowledge, five studies have investigated the association between HAP exposure and blood pressure among pregnant women, although these analyses differ in study design, types of solid fuels, and methods of HAP exposure assessment. Alexander et al. (2017) conducted a randomized controlled trial (RCT) for an ethanol cookstove intervention in pregnant women in Nigeria. Using an intention-to-treat (ITT) analysis, they found that the change in DBP over pregnancy was significantly different between ethanol users and control subjects, with an average of 2.8 mmHg higher DBP at each visit in control subjects than in ethanol users, while the SBP did not differ (Alexander et al., 2017). In Ghana, Quinn et al. investigated the cross-sectional association of personal exposure to CO and blood pressure among women during early- and mid-pregnancy and found a significant positive association between CO exposure and DBP: on average, each 1 ppm increase in CO was associated with 0.43 mmHg higher DBP (Quinn et al., 2016). They also estimated hourly personal CO exposures and measured ambulatory blood pressure in the same population to investigate the relationship between acute exposure to CO and the transient increases in blood pressure. The results suggested that peak CO exposure in the 2 h prior to blood pressure measurement was associated with elevations in both hourly SBP and DBP, as compared to blood pressure following lower CO exposures (Quinn et al., 2017). Two additional cross-sectional studies conducted in India (Agrawal and Yamamoto, 2015; Wylie et al., 2015) examined the relationship between solid fuel use and self-reported preeclampsia/eclampsia (pregnancy-induced hypertensive disorders) and hypertension, respectively. Agrawal and Yamamoto (2015) found that women living in households using solid fuels have two times higher likelihood of reporting preeclampsia/eclampsia symptoms than do those living in households using cleaner fuels; contrastingly, Wylie et al. (2015) found that compared to gas users, women using wood had on average lower mean blood pressure and the risk of hypertension was not significantly different between wood and gas users.

The gaps and inconsistencies in the current body of evidence highlight the need for additional investigation into the relationship between HAP exposure and blood pressure during pregnancy, with more accurate characterization of personal exposure and adequate adjustment of potential confounders. To our knowledge, no studies have examined the association between HAP exposure and gestational blood pressure using personal exposure assessment among rural women during early pregnancy who were exclusively using biomass in India. Here, we analyze data on personal exposure to PM_2.5_, BC, and CO and measures of gestational blood pressure at baseline for the 799 pregnant women enrolled in the Household Air Pollution Intervention Network (HAPIN) trial.

## 2 Methods

### 2.1 Study site and population

The current analysis utilizes baseline data collected from 799 pregnant women enrolled in the HAPIN trial at the India Intervention Research Center (IRC). The HAPIN Trial (ClinicalTrials.gov Identifier: NCT02944682) is an international multi-center study (India, Guatemala, Peru, and Rwanda) assessing the impact of a liquefied petroleum gas (LPG) cookstove and fuel intervention on health. The rationale, study design, and methods of the HAPIN trial have been described elsewhere (Clasen et al., 2020). Briefly, at each of the four IRCs, approximately 800 pregnant women from households using biomass fuel were identified and enrolled. Half of the households were randomly assigned to receive the intervention: a locally available LPG cookstove as well as 18-month supply of free LPG. The other half act as controls who continue to primarily use traditional cookstoves fueled with biomass. Mothers and children in both intervention and control groups were followed until the child reached one year old.

Here we report the cross-sectional association of blood pressure and HAP exposures among pregnant women at baseline in India. The HAPIN India IRC consists of two study sites in Tamil Nadu: Villupuram (VP) and Nagapattinam (NP). These study sites were chosen based on multiple criteria, including low rates of LPG penetration, low anticipated uptake of LPG among primary biomass fuel users over the 5 years of the trial, high exposure contrasts, and absence of major air pollution point sources. The hilly Villupuram study site is located at an altitude of approximately 10–165 m above sea level, while the Nagapattinam study site, a coastal area, is located at an average elevation between 10 and 50 m above the sea level (Clasen et al., 2020; Sambandam et al., 2020). Study sites were selected after thorough evaluation performed during a formative research period; detailed site selection methods and results in India IRC have been documented (Sambandam et al., 2020) (Thangavel et al., in preparation).

Pregnant women who participated in the study met a number of eligibility criteria: 1) confirmed pregnancy, 2) 18 to <35 years of age, 3) cook primarily with biomass stove, 4) live in the study area, 5) 9 to <20 weeks’ gestation with a singleton pregnancy confirmed by ultrasound, 6) continued pregnancy at the time of randomization and 7) agree to participate with informed consent (Clasen et al., 2020). Eligible pregnant women were excluded if they currently smoked, planned to move permanently outside the study area in the next 12 months, or planned to use a clean fuel stove predominantly in the near future (Clasen et al., 2020). Enrollment and data collection for HAPIN participants in the India IRC started in May 2018. Participants in the current analysis include pregnant women who had completed baseline assessment and randomization (n = 799). One participant was withdrawn due to an eligibility information error. Ethical approval for all HAPIN Trial activities in India IRC were obtained from the Institutional Ethics Committee at Sri Ramachandra Institute of Higher Education and Research (IEC-N1/16/JUL/54/49), Institutional Review Board of Emory University (00089,799), and the Indian Council of Medical Research–Health Ministry Screening Committee (5/8/4–30/(Env)/Indo-US/2016-NCD-I).

### 2.2 Exposure measurement

Details of the exposure assessment methods employed in the HAPIN trial are described elsewhere (Johnson et al., 2020). At baseline and before randomization, 24-h personal exposures to PM_2.5_, BC, and CO were assessed for all pregnant women. Real-time and integrated gravimetric personal PM_2.5_ exposures were measured using the Enhanced Children's MicroPEM™ (ECM) (RTI International, Research Triangle Park, USA). Real-time PM_2.5_ concentrations were estimated with ECM's nephelometer (light-scattering sensor); average 24-h exposures are estimated using gravimetric PM_2.5_ samples collected by drawing air through an impactor attached to a cassette containing a 15-mm Teflon® filter (PT15-AN-PF02; MTL Corporation) (Johnson et al., 2020). ECMs also log temperature, relative humidity, filter pressure drop, and tri-axial accelerometry. PM_2.5_ filters placed in ECMs were assessed for BC with SootScan™ Model OT21 Transmissometers (Magee Scientific) at Sri Ramachandra Institute of Higher Education and Research (SRIHER). Real-time personal exposures to CO were measured with EL-USB-300 monitors (Lascar Electronics), a lightweight, data-logging monitor the size of a large pen that runs on ½ AA batteries and has a sensing range between 0 and 300 ppm. Lascar monitors use an electrochemical cell to measure CO and record the concentration at the user-specified interval of 2.5 s.

Pregnant women wore samplers near their breathing zone. Samplers were placed in pockets of a customized vest co-designed by the participants and the SRIHER research team (Johnson et al., 2020; Sambandam et al., 2020). The women were instructed to always wear the vest during the 24-h monitoring period except when sleeping, bathing, or conducting activities not suitable for wearing the equipment. In those time periods, samplers were kept with the vest and hung on a stand close to participants. Compliance was evaluated using a series of questionnaires that recorded the field team's observations and responses from the participants to questions directly asking about wearing the monitors at the end of each sampling period. Samples were excluded if they did not meet the data quality criteria for sample duration, flow rate, and inlet pressure. The reported final PM_2.5_ exposures are gravimetric exposure concentrations. When gravimetric concentrations were not available – due to missing or damaged filters, or instrument failures, like flow faults – a device-specific corrected nephelometric average concentration was utilized (Johnson, Pillarisetti, Piedrahita, et al. in revision).

### 2.3 Blood pressure measurement

Resting blood pressure was measured in the right arm of seated pregnant women by a nurse or trained field worker as part of the baseline health assessment directly following the 24-h exposure monitoring period. Specifically, blood pressure was measured in triplicate with at least a 2-min resting period between repeat measurements using an automatic blood pressure monitor (model HEM-907XL; Omron®) (HAPIN study protocol v12.0, Clasen et al., 2020). The average of the three readings was used in the analysis. Blood pressure measurements were taken after ensuring that the pregnant woman had not smoked, consumed alcohol or a caffeinated beverage (coffee, tea, or Coca-Cola), or cooked using biomass in the 30 min prior to the measurement. If the participant had done any of above activities, she would be asked to refrain from these activities for 30 min before starting the blood pressure measurement (Clasen et al., 2020).

If a participant was found to have a SBP ≥ 140 mmHg and/or a DBP ≥ 90 mmHg, their blood pressure would be checked again during the same visit. If a participant had a SBP ≥ 140 mmHg and/or a DBP ≥ 90 mmHg on these two measurements, or if she had a SBP <80 mmHg or DBP <40 mmHg, the participant would be referred to the nearest health center or hospital to receive age-appropriate treatment. SBP values < 70 mmHg and DBP values < 35 mmHg were excluded as implausible. There were no implausible high vales.

### 2.4 Statistical analysis

All data analyses were conducted using R version 4.0.3 (R Core Team, 2021). Descriptive statistics were generated for key household and maternal characteristics, personal exposures, and blood pressures. Linear regression analyses were conducted to assess the association between HAP exposure (PM_2.5_, BC, and CO) and blood pressure. Average SBP and DBP of the three baseline measurements were the two outcome variables in separate models. Given the medium to high correlation between PM_2.5_ and BC exposures (Spearman's ρ [$\gamma_{s}$] = 0.77), PM_2.5_ and CO exposures ($\gamma_{s}$ = 0.60) and BC and CO exposures ($\gamma_{s}$ = 0.55) (Figure S1), exposure variables for different air pollutants were run separately in regression analyses. This resulted in three sets of multivariate linear models: SBP/DBP – PM_2.5_, SBP/DBP – BC, and SBP/DBP – CO. The regression equations (Eq. (1)) can be generalized as follows:

(1) $$\text{S}BP/DBP=\;\beta_{0}+\;\beta_{1}HAP+\sum \gamma Z+\;\varepsilon$$

where $\beta_{0}$ is the intercept; $\beta_{1}$ is the effect estimate for HAP exposure (PM_2.5_ or BC or CO) on SBP or DBP, holding other covariates constant; **Z** represents a matrix of potential covariates (described below); and ε is the model residual.

Covariates considered in the model are risk factors for (1) both personal exposure (to PM_2.5_, BC, or CO) and blood pressure levels or (2) for the blood pressure outcome. We identified covariates based on prior evidence and using a directed acyclic graph (DAG) generated using DAGitty v3.0 (Figure S2). We also considered univariate associations between covariates and SBP or DBP (Table S1).

Gestational age was determined by ultrasound at study enrollment. Maternal weight and height were recorded as part of baseline prenatal health exams, along with blood pressure measurements. Then, body mass index (BMI), defined as weight divided by the square of height, was calculated based on recorded measurements. Medical history and previous adverse pregnancy/birth events were obtained by questionnaire. Demographic and socioeconomic covariates, such as maternal age, mother's highest level of education, occupation, household size, and household wealth were recorded by questionnaires during baseline household visits. We calculated the relative household wealth using the EquityTool (Chakraborty et al., 2016) (https://www.equitytool.org/india/) specifically constructed for India based on the ownership of various household assets. Physical activity quartile was calculated based on the total MET (Metabolic Equivalent of Task) minutes per week using the World Health Organization Global Physical Activity Questionnaire (GPAQ). A MET is the ratio of the rate of energy expended during an activity to the rate of energy expended at rest (Physical activity guidelines for Americans, 2008). Factors that may influence blood pressure level (e.g., weekday/weekend and morning/afternoon of the blood pressure measurement, consumption of caffeinated beverage) and personal exposure (e.g., season, passive smoking, incense, and mosquito coils burning etc.) were also recorded by questionnaire.

We retained covariates in the final model if they changed the parameter estimate for the association between exposure and health endpoints by more than 10% based on adding and removing covariates individually in univariate analyses. The final adjusted model contained covariates for age, BMI, mother's highest level of education, household wealth index at national quintile, gestational age, and season of measurement.

Personal exposure to PM_2.5_, BC, and CO were natural log transformed in regression analyses to meet the assumptions of regression modeling, specifically to avoid nonlinearity, as diagnosed in partial residual plots and other standard regression diagnostics. We also examined categorical (quartile) personal exposures in all models. As sensitivity analyses, we fit a generalized additive model (GAM) to assess the potential nonlinear trends between HAP exposures and SBP/DBP. A smooth function was applied to exposure variables with an optimized smoothing parameter via restricted maximum likelihood. Various values of the basis dimension (k) were tested with gam.check(). Additionally, we tested potential effect modification by study site in the log linear models by including an interaction term between PM_2.5_/BC/CO exposure and study site. Finally, we reproduced the analysis after excluding the highest 1% of exposure observations.

## 3 Results

***Baseline Characteristics****.* In Table 1, we summarize descriptive characteristics of pregnant women (n = 799) enrolled in the HAPIN trial in India. The mean (SD) gestational age of pregnant women was 16.1 (3.0) weeks at baseline. None of the participants reported previously being hypertensive or diabetic. Nearly half of participants were pregnant for the first time (49%). Among those who had been pregnant before, 12 (2%) had a history of preterm birth, 84 (11%) experienced spontaneous abortion, and 15 (2%) had stillbirths. The mother's level of education and occupation were distinct by study sites, with higher education and more mothers working from home in Nagapattinam. More than half (52%) of the women reported no formal education or incomplete primary school at the Villupuram site, while only 20% of the women reported the same in Nagapattinam (Table S2). The majority (84%) of the women were agricultural workers in Villupuram, while in Nagapattinam, nearly all (97%) participants reported no work outside of the home (Table S2). Wood was the sole biomass fuel used in the households; 95% of the pregnant women were primary cooks. Although all pregnant women were non-smokers, about one-third (32%) of households had one or more family members who smoke at home. No participant reported being on high blood pressure medication at the baseline.

**Table 1:** Baseline characteristics of pregnant women at the HAPIN India IRC.

	India IRC (N = 799)
Maternal age at baseline (years), mean (SD), min - max	24.0 (3.8) 18.1–34.8
Gestational age at baseline (weeks), mean (SD), min-max	16.1 (3.0) 9.6–24.9
BMI (kg/m^2^) at baseline, mean (SD), min-max	19.7 (3.2) 13.3–37.6
Nulliparity, n (%)	
Yes	459 (57%)
No	340 (43%)
Mother's highest education level, n (%)	
No formal education or Primary school incomplete	258 (36%)
Primary school complete or Secondary school incomplete	227 (28%)
Secondary school complete or Vocational or Some college or university	287 (36%)
Mother's occupation outside the home, n (%)	
Agriculture/farming	338 (42%)
Service/commercial	4 (1%)
No work outside of the home	432 (54%)
Other	25 (3%)
History of preterm birth, Yes (%)	12 (2%)
History of spontaneous abortion, Yes (%)	84 (11%)
History of stillborn, Yes (%)	15 (2%)
Previous history of hypertension, Yes (%)	0
Previous history of diabetes, Yes (%)	0
Physical activity (total MET min/week), mean (SD)	
Quartile 1 (<1680)	761.0 (336.0)
Quartile 2 (≥ 1680 and < 5040)	2749.0 (925.0)
Quartile 3 (≥ 5040 and < 6720)	5229.0 (348.0)
Quartile 4 (≥ 6721)	9401.0 (4187.0)
Household size, mean (SD), min - max	3.8 (1.5) 1.0–10.0
Household wealth at national quintiles, n (%)	
Lowest (1)	179 (22%)
Second lowest (2)	401 (50%)
Medium (3)	176 (22%)
Second highest (4)	43 (5%)
Primary fuel type, n (%)	
Wood	799 (100%)
Cook in the past 24 h, n (%)	
Pregnant women	767 (96%)
Others	32 (4%)
Someone in household smokes, n (%)	
Yes	253 (32%)
No	546 (68%)

***Exposure****.* Compliance with the exposure monitoring is considered good. Only 22 (3%) participants were observed not wearing the equipment nor having the equipment nearby when field workers arrived at the household. Among all baseline personal HAP exposure measurements, 89% (N = 715) of pregnant women had valid PM_2.5_ samples, 87% (N = 699) had valid BC measurements and 93% (N = 745) had valid CO samples. Table 2 provides the descriptive statistics of valid personal PM_2.5_, BC and CO exposures of pregnant women. Distributions of exposures are visualized in Figure S3. During the baseline period, the 24-h PM_2.5_ exposures of 86% of the participants were above the World Health Organization's Interim Target 1 for annual mean concentrations of 35 μg/m^3^. The PM_2.5_, BC, and CO personal exposures were all significantly higher in Villupuram than in Nagapattinam. The median (IQR) 24-h personal exposure to PM_2.5_ was 75.5 (82.2) μg/m^3^ [Villupuram: 99.2 (108.0) μg/m^3^; Nagapattinam: 59.4 (59.5) μg/m^3^]. The overall median (IQR) of 24-h personal exposure to BC was 9.6 (10.7) μg/m^3^ [Villupuram: 13.7 (12.5) μg/m^3^; Nagapattinam: 7.0 (7.3) μg/m^3^]. For 24-h CO exposure, the overall median (IQR) level was 0.8 (1.8) ppm [Villupuram: 1.1 (2.3) ppm; Nagapattinam: 0.6 (1.2) ppm]. The higher 24-h HAP exposures at the Villupuram site were likely due to more fuel consumption, longer stove use time, differences in kitchen configurations, and other indoor and outdoor exposure sources. The 24-h personal exposures to PM_2.5_, BC and CO are also examined after removing the highest 1% of the measurements. The results are presented in Table S3.

**Table 2:** Summary of 24-h personal exposures to PM_2.5_, BC, and CO among pregnant women using biomass at the HAPIN India IRC (based on valid samples only).

		N	India IRC
PM_2.5_ exposure (μg/m^3^)	Median (IQR), Mean [Range]	715	75.5 (82.2), 115.2 [9.4, 2100.0]
BC exposure (μg/m^3^)	Median (IQR), Mean [Range]	699	9.6 (10.7), 12.9 [0.6, 102.7]
CO exposure (ppm)	Median (IQR), Mean [Range]	745	0.8 (1.8), 1.8 [0, 46.9]

***Blood Pressure***. All participants had valid blood pressure measurements at baseline and were largely normotensive (SBP <120 and DBP <80) (95%). The mean (SD) of SBP was 104.5 (9.1) mmHg and the mean (SD) of DBP was 61.5 (7.6) mmHg (Table 3). Distributions of baseline SBP and DBP by study sites are visualized in Figure S4. 13 pregnant women (2%) were considered as at hypertension stage 1, defined as SBP between 130 and 139 mmHg or DBP between 80 and 89 mmHg. Two individuals (<1% of the participants) were categorized as overly hypertensive, defined as SBP ≥ 140 mmHg or DBP ≥ 90 mmHg. The categorizations of normal blood pressure and of different stages of elevated blood pressure were not different between the two study sites.

**Table 3:** Blood pressure measurement summary for pregnant women using biomass at the HAPIN India IRC.

	India IRC (N = 799)
SBP (mmHg), mean (SD)	104.5 (9.1)
DBP (mmHg), mean (SD)	61.5 (7.6)
Blood pressure category^a^^.^, n (%)	
Normal (SBP <120 and DBP <80)	758 (95%)
Elevated (SBP in 120–129 and DBP <80)	26 (3%)
High blood pressure (hypertension) stage 1 (SBP in 130–139 or DBP in 80–89)	13 (2%)
High blood pressure (hypertension) stage 2 (SBP ≥ 140 or DBP ≥ 90)	2 (<1%)

a American Heart Association, American College of Cardiology (AHA/ACC) blood pressure classification.

***Statistical Analysis****.* Regression analyses for each HAP-gestational blood pressure pair were restricted to participants with valid exposure and blood pressure measurements. This resulted in a sample size of 715 for PM_2.5_-gestational blood pressure analysis, 699 for BC-gestational blood pressure analysis, and 730 for CO-gestational blood pressure analysis (15 CO samples with values of 0 were excluded). The adjusted estimates for the effects of PM_2.5,_ BC, and CO exposure on SBP and DBP are presented in Table 4. Complete unadjusted and adjusted model outputs can be found in supplemental Table Tables S4-S7.

**Table 4:** Adjusted association between personal PM_2.5_ (N = 715), BC (N = 699) and CO (N = 730) and gestational blood pressure. Results are presented in estimate and 95% confidence interval.

	Model Type	Estimate	p-value	95% CI	AIC
**Systolic Blood Pressure**					
PM_2.5_	Log linear	0.4411	0.2985	(-0.3913, 1.2734)	5177
	Exposure Quartiles [Ref. Quartile 1, <47.7 μg/m^3^]				
	Quartile 2, <75.5 μg/m^3^	−0.4036	0.6772	(-2.306, 1.4989)	5180
	Quartile 3, <129.9 μg/m^3^	0.3044	0.7535	(-1.5983, 2.2071)	
	Quartile 4, ≥ 129.9 μg/m^3^	0.7824	0.4201	(-1.1217, 2.6865)	
BC	Log linear	0.33	0.4392	(-0.507, 1.167)	5066
	Exposure Quartiles [Ref. Quartile 1, <5.5 μg/m^3^]				
	Quartile 2, <9.6 μg/m^3^	0.3304	0.7338	(-1.5764, 2.2371)	5068
	Quartile 3, <16.2 μg/m^3^	1.0504	0.2849	(-0.8766, 2.9775)	
	Quartile 4, ≥ 16.2 μg/m^3^	1.1592	0.242	(-0.7845, 3.1028)	
CO	Log linear	0.3903	0.0604	(-0.0171, 0.7976)	5259
	Exposure Quartiles [Ref. Quartile 1, <0.4 ppm]				
	Quartile 2, <0.9 ppm	1.6371	0.0785	(-0.1868, 3.461)	5261
	Quartile 3, <2.1 ppm	1.9632	0.0342	(0.1464, 3.78)	5261
	Quartile 4, ≥ 2.1 ppm	1.1915	0.2019	(-0.6398, 3.0228)	5261
***Diastolic Blood Pressure***					
PM_2.5_	Log linear	0.6528	0.0584	(-0.0231, 1.3287)	4879
	Exposure Quartiles [Ref. Quartile 1, <47.7 μg/m^3^]				
	Quartile 2, <75.5 μg/m^3^	−0.5609	0.4748	(-2.101, 0.9791)	4878
	Quartile 3, <129.9 μg/m^3^	1.032	0.1888	(-0.5082, 2.5722)	
	Quartile 4, ≥ 129.9 μg/m^3^	1.5151	0.054	(-0.0263, 3.0565)	
BC	Log linear	0.5038	0.1487	(-0.1804, 1.1881)	4784
	Exposure Quartiles [Ref. Quartile 1, <5.5 μg/m^3^]				
	Quartile 2, <9.6 μg/m^3^	0.2138	0.7876	(-1.344, 1.7717)	4786
	Quartile 3, <16.2 μg/m^3^	1.2862	0.1092	(-0.2882, 2.8606)	
	Quartile 4, ≥ 16.2 μg/m^3^	1.2893	0.1114	(-0.2987, 2.8773)	
CO	Log linear	0.3595	0.0365	(0.0226, 0.6963)	4981
	Exposure Quartiles [Ref. Quartile 1, <0.4 ppm]				
	Quartile 2, <0.9 ppm	1.1015	0.1515	(-0.4047, 2.6078)	4981
	Quartile 3, <2.1 ppm	1.6005	0.0366	(0.1002, 3.1009)	
	Quartile 4, ≥ 2.1 ppm	2.0838	0.007	(0.5714, 3.5961)	

Note: All models adjusted for age, BMI, mother's highest level of education, household wealth index, gestational age, and season.

In adjusted models, we found that pregnant women with PM_2.5_ exposures in the highest quartile had a marginal significant association with DBP (1.50 mmHg, 95% CI: 0.03, 3.06) compared to those who had PM_2.5_ exposures in the lowest quartile. There was also some evidence of a log-linear trend for DBP (0.65 mmHg, 95% CI: 0.02, 1.33). No evidence of an association was observed for BC exposure and gestational blood pressure. For the CO-gestational blood pressure relationship, adjusted mean DBP was 0.36 mmHg higher (95% CI: 0.02, 0.70) per unit increase in natural log-transformed personal CO exposure. Results were stronger with CO exposure levels in the 3rd and 4th quartile, compared to those with exposure levels in the lowest quartile.

In sensitivity analyses, we used GAMs to assess the PM_2.5_-gestational blood pressure, BC-gestational blood pressure, and CO-gestational blood pressure relationships, using a smooth term for exposures while controlling for other covariates. No model showed significance with a smoothed exposure term. Figure S5 illustrates the associations between SBP/DBP and exposures to PM_2.5_/BC/CO. Although the associations between BC/CO and DBP appeared to be some deviations from linearity at high levels of exposure, very few observations were at these levels and the confidence intervals were wide. The result suggests that there is some complexity in the relationship between BC/CO and DBP; however, the shape and direction of those effects is uncertain. Outputs of GAMs analysis are provided in Supplementary Information.

Furthermore, we assessed effect modification in the adjusted log-linear regression models by adding an interaction term between the exposure variable and the study site indicator variable. No model had a significant interaction term. Additionally, a stratified analysis by study site was conducted with all adjusted log-linear regression models. No significant association was observed between HAP exposure and gestational blood pressure at the study site level. Finally, excluding the highest 1% of exposure observations did not appreciably change the associations between PM_2.5_/CO and gestational blood pressure. However, a marginally significant association was observed for BC and DBP in both log linear exposure and categorical exposure models after removing the highest 1% of exposure measurements (Table S9).

## 4 Discussion

This is the first study to examine the relationship between HAP and gestational blood pressure using quantitively measured personal exposures and blood pressure among pregnant Indian women using solid fuels in their early pregnancy. This cohort of pregnant women does not have common risk factors for elevated blood pressure or hypertension; they are predominantly young, with relatively low BMI and socioeconomic status. However, we found that SBP and DBP were higher in pregnant women exposed to higher levels of PM_2.5_ and CO, though only the results for the association between CO and DBP reached conventional statistical significance (α ≤ 0.05). We observed a positive association between CO and DBP among the entire study cohort; the effect was stronger in pregnant women with higher (in 3rd [≥ 0.9 and < 2.1 ppm] and 4th quartile [≥ 2.1 and ≤ 46.9 ppm]) CO exposures. As for the relationship between PM_2.5_ and DBP, we only saw a borderline significant positive association among all participants. The effect was slightly stronger among pregnant women with PM_2.5_ exposures in the highest quartile (≥ 129.9 and ≤ 2100 μg/m^3^) compared to those who had exposures in the lowest quartile (≥ 9.4 and < 47.7 μg/m^3^).

Our finding supports evidence from prior studies conducted in pregnant women. In a similar baseline assessment of a cookstove intervention study with Ghanaian pregnant women of early gestation (average 16 weeks), a significant positive association was also found between CO exposure and DBP (an increase of 0.43 mmHg DBP for each 1 ppm elevation in CO exposure), while a non-significant positive trend was observed for SBP (Quinn et al., 2016). In another intervention study, results from an ITT analysis of an ethanol cookstove intervention in pregnant Nigerian women suggested that DBP levels were significantly higher in control subjects using kerosene/firewood cookstoves; however, the SBP level did not differ between the intervention and control groups (Alexander et al., 2017). The magnitude of the association observed in our study population (Table S5) is smaller in scale than the associations between CO and DBP that have been reported in Ghana, although the mean CO exposure in our cohort (2.0 ppm) was slightly higher than that of the Ghanaian cohort of pregnant women (1.6 ppm) (Quinn et al., 2016). This smaller effect of CO on blood pressure may be due to the younger age (24.0 ± 3.8 vs. 27.3 ± 7.1) and lower BMI of our participants (19.7 ± 3.2 vs. 23.3 ± 3.3). Additionally, the mean SBP and DBP are both lower in our study (SBP: 104.5 ± 9.1 mmHg; DBP: 61.5 ± 7.6 mmHg), compared to those in Ghana (SBP: 105.5 ± 10.2 mmHg; DBP: 63.2 ± 7.9 mmHg) (Quinn et al., 2016, 2017) and Nigeria (SBP: 110.4 ± 11.9 [intervention] and 111.3 ± 10.8 mmHg [control]; DBP: 69.2 ± 9.5 mmHg [intervention] and 70.0 ± 10.3 [control]) at baseline (Alexander et al., 2017).

Our results are not entirely consistent with findings from similar studies conducted in non-pregnant women. Unlike the strong association observed between personal exposure to PM_2.5_ and blood pressure in non-pregnant Chinese women (Baumgartner et al., 2011; 2018, 2014; Chen et al., 2020), we did not find a significant PM_2.5_–gestational blood pressure association across our entire study cohort. Among all women enrolled, they found a 1-log-μg/m^3^ increase in PM_2.5_ exposure was associated with 2.2 mmHg higher SBP (95% CI: 0.8, 3.7) and 0.5 mmHg higher DBP (95% CI: 0.4, 1.3) and the estimated effects were stronger among women >50 years of age (Baumgartner et al., 2011; 2018, 2014). Also, no evidence of association was observed for BC exposure and gestational blood pressure in our main analysis, though some evidence suggested a positive BC-DBP association after removing the highest 1% of BC exposures. However, a stronger association between BC (compared to PM_2.5_) and blood pressure (a 1-log-μg/m^3^ increase in BC was associated with 4.3 mmHg higher SBP) was observed in Baumgartner et al. (2018, 2014). In Honduras, Young et al. (2019) find no significant association between personal exposures to PM_2.5_ or BC and BP; in Nicaragua, Clark et al. (2011) only observed a significant relationship between personal CO exposure and SBP in overweight participants; while in India, results from Norris et al. (2016) suggested increases in personal exposure to BC were actually associated with small decreases in DBP.

The varying direction and scale of the effects of HAP on blood pressure in the above studies compared to ours may be a result of the differences in the type of solid fuels being used, cooking practices, age, BMI, basal blood pressure levels, and, more importantly, pregnancy, a factor known to influence blood pressure. In clinically healthy pregnant women, blood pressure steadily decreases up to the middle of gestation and increases up to the day of delivery, with final blood pressure values similar to those found early in pregnancy in the same women (Hermida et al., 2000). However, after controlling for gestational age in our analysis, we still observed strong associations between CO and DBP among highly exposed participants and across the entire group of study participants. These findings, together with the largely consistent evidence from other studies conducted in pregnant women, provide evidence confirming an association between HAP exposure and gestational blood pressure – particularly the influence on DBP.

As other studies have noted, the observed small size of the blood pressure changes induced by HAP exposure are not likely to have significant clinical relevance for individuals, especially among our young and low risk cohort, However, even small changes in blood pressure at the population level can translate into significant health burdens globally, given the large number of populations exposed to HAP on a daily basis. For example, depending on age, a 5 mmHg lower than usual DBP was associated with 19%–50% lower total stroke risk and 11%–41% lower ischemic heart disease risk (Asia Pacific Cohort Studies Collaboration, 2003). In addition, blood pressure levels are different between complicated and uncomplicated pregnancies as early as in the first trimester of gestation (Hermida et al., 2000). Understanding the association between HAP exposure and blood pressure in early- and mid-pregnancy may have important implications for blood pressure monitoring and management among pregnant women, as continuous elevation in blood pressure may develop into gestational hypertension and preeclampsia and could contribute to the risk of developing hypertension later in life (Asayama and Imai, 2018).

An important limitation of this study is its cross-sectional design. Burning solid fuels is a long-term behavior and air pollution-triggered changes in blood pressure can occur both acutely and over long periods of time, such as days or months (Brook et al., 2009). A 24-h HAP exposure measurement and a single blood pressure measurement may not represent true average exposures or blood pressures, and our analysis cannot establish the duration of air pollution effects on blood pressure (Baumgartner et al., 2018). The observational study design also has the inherent challenge of residual confounding (Díaz et al., 2007; Smith et al., 2011). Future analysis of a longitudinal, repeat measures exposure-response relationship over the entire pregnancy under a clean energy intervention regime in this population may facilitate the evaluation of longer-term changes in HAP exposure and gestational blood pressure.

We also did not have data on some important potential covariates that might confound or modify the association between HAP exposure and gestational blood pressure, such as sodium intake and ambient temperature (Brook et al., 2011; Clark et al., 2019; Quinn et al., 2016). However, in our study area, the ambient temperature does not vary considerably within the same season (timeanddate.com) and we have controlled for season in all HAP-gestational blood pressure models.

Despite these limitations, our study has several notable methodological strengths and innovations. To our knowledge, this is the first study in India that has established the association between HAP and blood pressure using quantitively measured personal exposure and blood pressure among pregnant women. The extensive measurements in such a large cohort permit us to understand the ‘usual’ daily HAP exposures of pregnant women using solid fuels, the blood pressure distribution during early- and mid-pregnancy, and the relationships between the two. Furthermore, we measured PM_2.5_, BC, and CO, major constituents of HAP from solid fuels burning, simultaneously. This allows us to understand the relationship between these different household air pollutants and examine their association with blood pressure individually.

Elevated blood pressure is recognized as one of the most important independent risk factors for cardiovascular disease in India and presents a special concern for pregnant women. Our study contributes to the limited evidence on the association between HAP and blood pressure among pregnant women and suggests that interventions that reduce HAP exposures may improve blood pressure, and subsequently, reduce the incidence of gestational hypertension that could contribute to the risk of adverse pregnancy outcomes and hypertension later in life. More broadly, our characterization of the unique HAP exposure profiles among women using solid fuels in South India may be useful to better understand the magnitude of health effects attributable to air pollution in this region and inform interventions and policies aimed at reducing HAP exposure and associated health effects in India and other developing countries.

## Declaration of competing interest

The authors declare that they have no known competing financial interests or personal relationships that could have appeared to influence the work reported in this paper.
