# Supplementary material for: Association between personal exposure to household air pollution and gestational blood pressure among women using solid cooking fuels in rural Tamil Nadu, India
Source: Environ Res. 2022 May 15;208:112756. doi: 10.1016/j.envres.2022.112756 (PMC8935388; doi:10.1016/j.envres.2022.112756)
Supplement: Multimedia component 1 [file mmc1.docx]

**Supplementary Information**

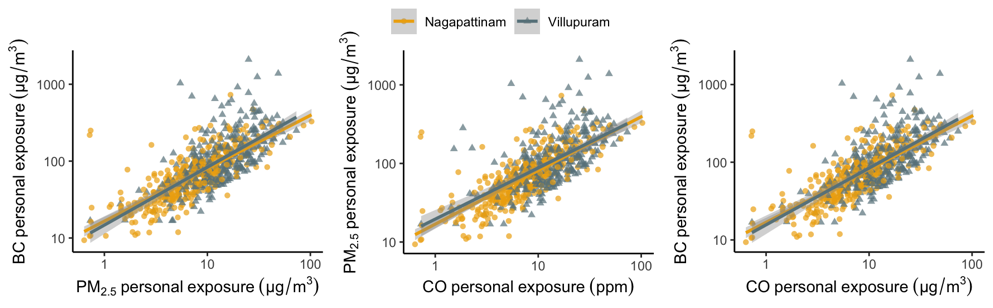


**Figure S1**. Relationship between PM2.5 and BC, PM2.5 and CO, and BC and CO in India IRC (upper) and by study district (lower). $\gamma_{s}$ indicates the Spearman’s $\rho$. (Based on valid samples)


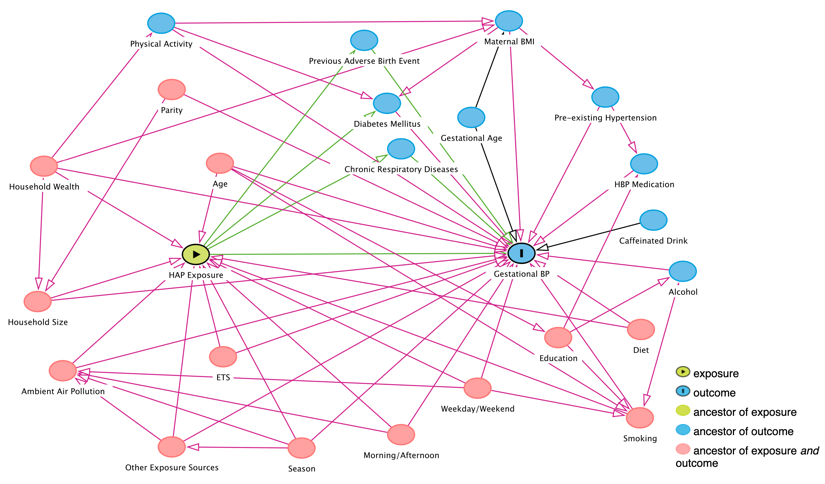


**Figure S2**. DAG to guide the selection of confounders for the association between household air pollution exposure and gestational blood pressure. Causal path (green line); Biasing path (red line). *Minimal sufficient adjustment sets for estimating the total effect of HAP Exposure on Gestational BP based on this DAG: age, ambient air pollution, diet, ETS, household size, household wealth, morning/afternoon, other exposure sources, season, smoking, weekday/weekend.*


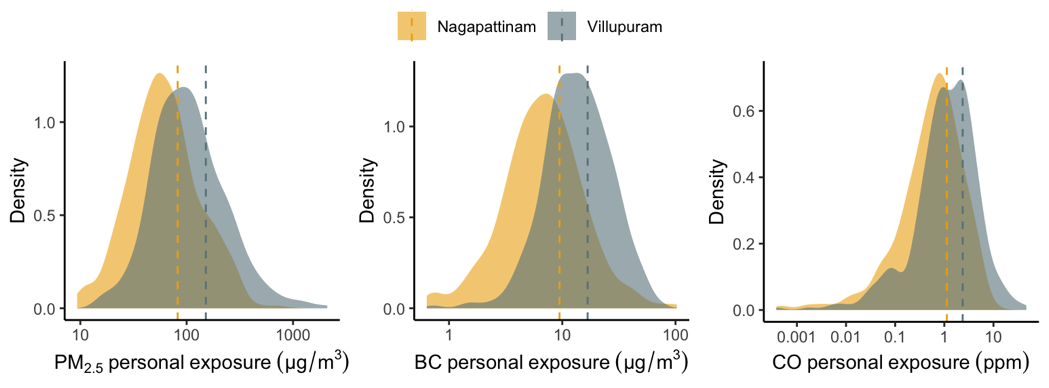


**Figure S3**. Distribution of 24-hour personal exposure to PM_2.5_, BC and CO at baseline by study sites. Dashed lines are the mean exposure values by site; x-axes are log_10_-transformed.


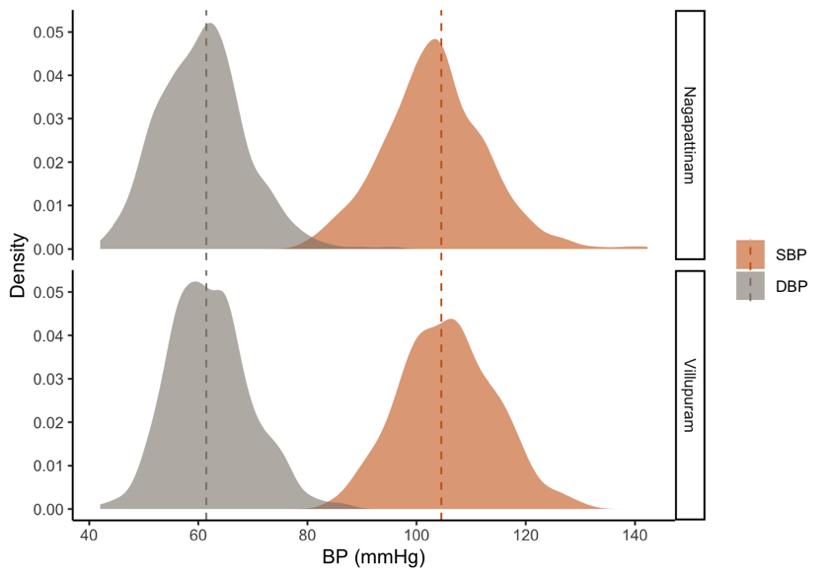


**Figure S4.** Distribution of baseline SBP and DBP by study districts. Dashed lines indicate the mean BP values.

**
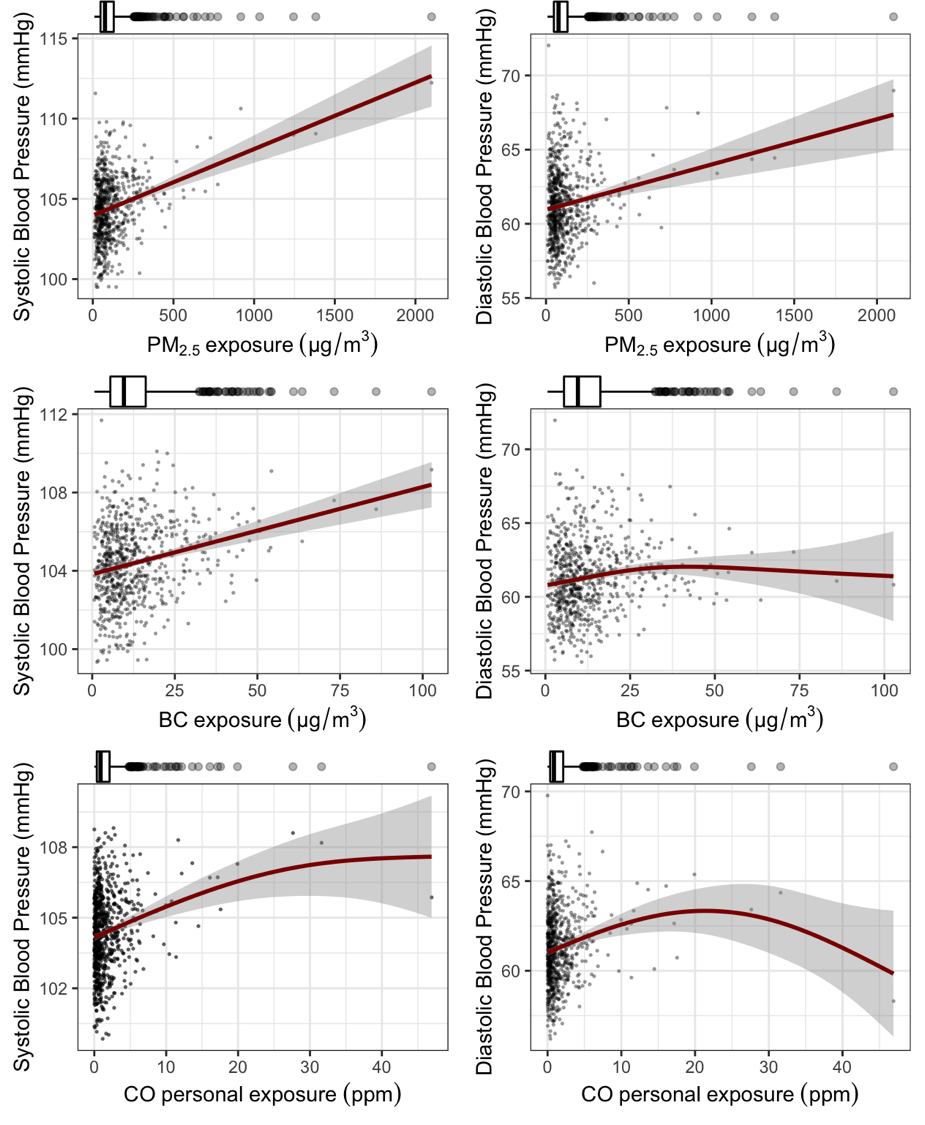
**

**Figure S5.** HAP-GBP relationship (dark red solid line) and associated 95% confidence interval (shade) based on generalized additive models (GAMs) with thin plate regression splines with 4 degrees of freedom, adjusting for age, BMI, mother’s highest level of education, household wealth index, gestational age, and season.


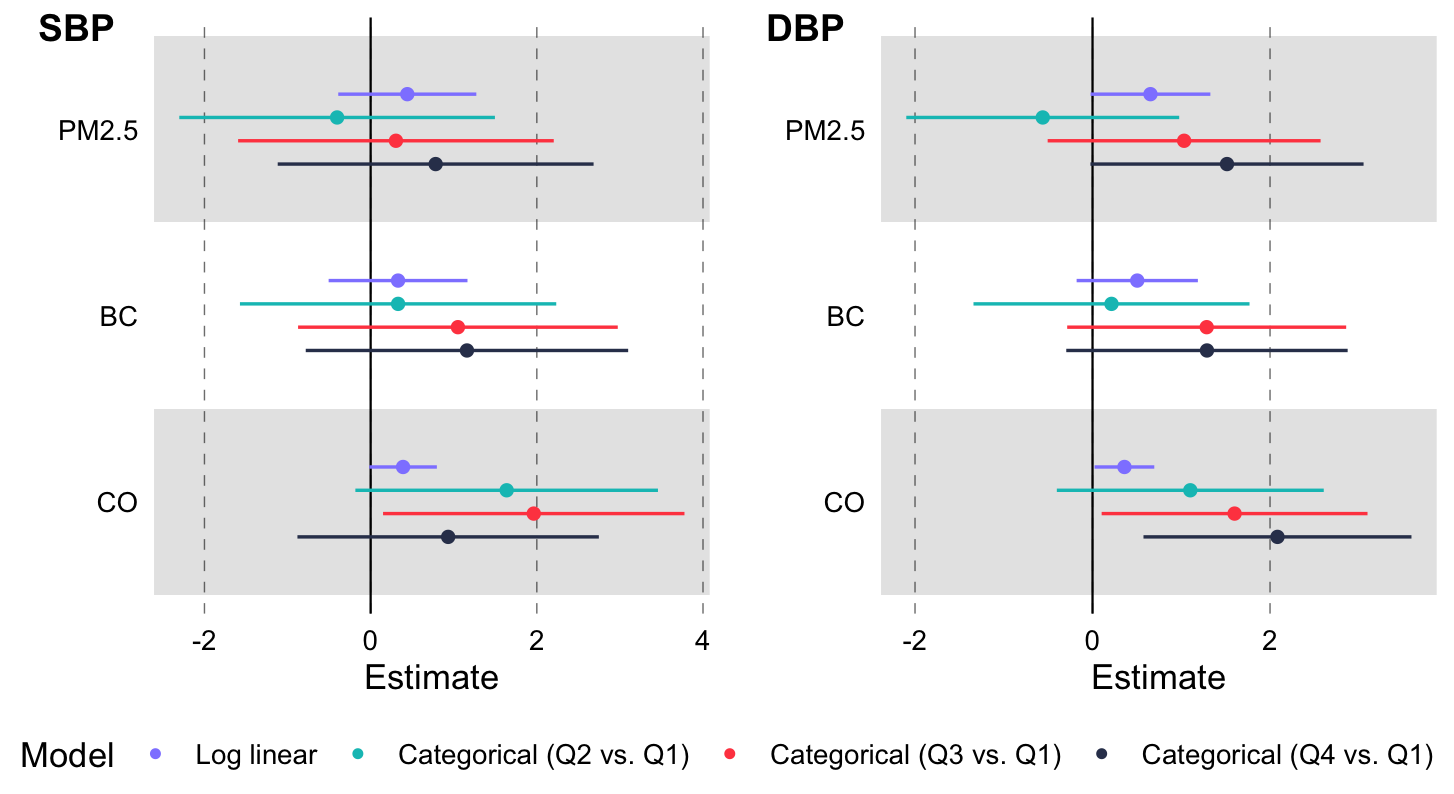


**Figure S6.** Forest plot of adjusted association between personal PM_2.5_, BC, and CO and gestational blood pressure. Results are presented in estimate (dots) and 95% confidence interval.

**Table S1**. Univariate association of maternal and household characteristics with SBP and DBP

| **Variable** | **Coefficient** | **SE** | **p-value** |
| --- | --- | --- | --- |
| **SBP (n = 799)** | | | |
| Maternal age at baseline in years | -0.05 | 0.08 | 0.588 |
| BMI | 0.25 | 0.10 | 0.0116 * |
| GBP measured on weekends [Ref. No] | -0.03 | 0.77 | 0.969 |
| GBP measured in the morning [Ref. No] | 1.33 | 0.78 | 0.0874 . |
| Household size | 0.08 | 0.21 | 0.706 |
| Mother’s education [Ref. No formal education/primary school incomplete] | | | |
| Primary school complete/Secondary school incomplete | -1.01 | 0.80 | 0.207837 |
| Secondary school complete/Vocational or Some college/University | -2.50 | 0.76 | 0.000978 *** |
| Household wealth at national quintile [Ref. Lowest (1)] |  |  |  |
| Second lowest (2) | -0.89 | 0.82 | 0.2744 |
| Medium (3) | -1.79 | 0.96 | 0.0635 . |
| Second highest (4) | -3.32 | 1.54 | 0.0316 * |
| Physical activity (total MET-min/week) [Ref. Quartile 1] |  |  |  |
| Quartile 2 | -0.84 | 0.96 | 0.3821 |
| Quartile 3 | -1.88 | 1.06 | 0.0777 . |
| Quartile 4 | -1.46 | 0.90 | 0.1045 |
| Total # of pregnancy [Ref. 1] |  |  |  |
| 2 | -0.38 | 0.72 | 0.603 |
| 3+ | -0.72 | 0.90 | 0.428 |
| Nulliparity [Ref. No] | -0.21 | 0.65 | 0.75 |
| Gestational age at baseline in weeks | -0.14 | 0.11 | 0.191 |
| Mother’s occupation [Ref. Agriculture] |  |  |  |
| Commercial | -0.26 | 4.54 | 0.955104 |
| Household | -2.37 | 0.66 | 0.000315 *** |
| Other | -1.00 | 1.87 | 0.593770 |
| Season [Ref. Winter (Jan. – Feb.)] |  |  |  |
| Summer (Mar. – May) | -2.43 | 1.16 | 0.0373 * |
| Monsoon (Jun. – Dec.) | -0.82 | 1.04 | 0.4320 |
| **DBP (n = 799)** | | | |
| Maternal age at baseline in years | 0.11 | 0.07 | 0.12 |
| BMI | 0.44 | 0.08 | 2.41e-07 *** |
| GBP measured on weekends [Ref. No] | -1.02 | 0.65 | 0.115 |
| GBP measured in the morning [Ref. No] | 0.29 | 0.65 | 0.657 |
| Household size | -0.09 | 0.18 | 0.618 |
| Mother’s education [Ref. No formal education/primary school incomplete] | | | |
| Primary school complete/Secondary school incomplete | -0.49 | 0.68 | 0.469 |
| Secondary school complete/Vocational or Some college/University | -0.95 | 0.64 | 0.136 |
| Household wealth at national quintile [Ref. Lowest (1)] |  |  |  |
| Second lowest (2) | -0.32 | 0.69 | 0.6440 |
| Medium (3) | -1.37 | 0.81 | 0.0912 . |
| Second highest (4) | -0.48 | 1.30 | 0.7085 |
| Physical activity (total MET min/week) [Ref. Quartile 1] |  |  |  |
| Quartile 2 | -0.90 | 0.81 | 0.2646 |
| Quartile 3 | -1.65 | 0.89 | 0.0644 . |
| Quartile 4 | 1.11 | 0.76 | 0.1437 |
| Total # of pregnancy [Ref. 1] |  |  |  |
| 2 | -0.24 | 0.60 | 0.6934 |
| 3+ | -1.32 | 0.78 | 0.0805 . |
| Nulliparity [Ref. No] | -0.15 | 0.55 | 0.782 |
| Gestational age at baseline in weeks | -0.35 | 0.09 | 7.94e-05 *** |
| Mother’s occupation [Ref. Agriculture] |  |  |  |
| Commercial | -0.39 | 3.84 | 0.9190 |
| Household | -1.07 | 0.55 | 0.0531 . |
| Other | -0.63 | 1.58 | 0.6902 |
| Season [Ref. Winter (Jan. – Feb.)] |  |  |  |
| Summer (Mar. – May) | -1.80 | 0.97 | 0.0649 . |
| Monsoon (Jun. – Dec.) | 0.32 | 0.87 | 0.7057 |

***Note:***

1. Signifiance codes: ‘***’ 0.001 ‘**’ 0.01 ‘*’ 0.05 ‘.’ 0.1

**Table S2.** Baseline characteristics of pregnant women at the HAPIN India IRC, by study districts

|  | **India IRC**  **(N = 799)** | **Villupuram**  **(N = 400)** | **Nagapattinam**  **(N = 399)** |
| --- | --- | --- | --- |
| Maternal age at baseline (years), mean (SD), min - max | 24.0 (3.8)  18.1 – 34.8 | 23.1 (3.4)  18.1 – 34.2 | 24.8 (4.0)  18.1 – 34.8 |
| Gestational age at baseline (weeks), mean (SD), min-max | 16.1 (3.0)  9.6 – 24.9 | 15.5 (3.0)  9.6 – 22.4 | 16.6 (3.0)  9.6 – 24.9 |
| BMI (kg/m^2^) at baseline, mean (SD), min-max | 19.7 (3.2)  13.3 – 37.6 | 18.8 (2.3)  13.7 – 31.2 | 20.7 (3.6)  13.3 – 37.6 |
| Nulliparity, n (%) |  |  |  |
| Yes | 459 (57%) | 189 (47%) | 270 (68%) |
| No | 340 (43%) | 211 (53%) | 129 (32%) |
| Mother’s highest education level, n (%) |  |  |  |
| No formal education or Primary school incomplete | 258 (36%) | 207 (52%) | 78 (20%) |
| Primary school complete or Secondary school incomplete | 227 (28%) | 100 (25%) | 127 (32%) |
| Secondary school complete or Vocational or Some college or university | 287 (36%) | 93 (23%) | 194 (48%) |
| Mother’s occupation outside the home, n (%) |  |  |  |
| Agriculture/farming | 338 (42%) | 337 (84%) | 1 (<1%) |
| Service/commercial | 4 (1%) | 3 (1%) | 1 (<1%) |
| No work outside of the home | 432 (54%) | 45 (11%) | 387 (97%) |
| Other | 25 (3%) | 15 (4%) | 10 (3%) |
| History of preterm birth, Yes (%) | 12 (2%) | 8 (2%) | 4 (1%) |
| History of spontaneous abortion, Yes (%) | 84 (11%) | 32 (8%) | 52 (13%) |
| History of stillborn, Yes (%) | 15 (2%) | 7 (2%) | 8 (2%) |
| Previous history of hypertension, Yes (%) | 0 | 0 | 0 |
| Previous history of diabetes, Yes (%) | 0 | 0 | 0 |
| Physical activity (total MET min/day), mean (SD) |  |  |  |
| Quartile 1 | 109 (48.1) | 110 (46.0) | 86.3 (88.3) |
| Quartile 2 | 393 (132) | 353 (133) | 449 (110) |
| Quartile 3 | 747 (49.7) | 788 (72) | 736 (36) |
| Quartile 4 | 1343 (598) | 1878 (777) | 1077 (152) |
| Household size, mean (SD), min - max | 3.8 (1.5)  1.0 – 10.0 | 3.7 (1.6)  1.0 – 10.0 | 3.8 (1.4)  2.0 – 9.0 |
| Household wealth at national quintiles, n (%) |  |  |  |
| Lowest (1) | 179 (22%) | 93 (23%) | 86 (21.5%) |
| Second lowest (2) | 401 (50%) | 232 (58%) | 169 (42.5%) |
| Medium (3) | 176 (22%) | 71 (18%) | 105 (26%) |
| Second highest (4) | 43 (5%) | 4 (1%) | 39 (10%) |
| Primary fuel type, n (%) | Wood (100%) | Wood (100%) | Wood (100%) |
| Cook in the past 24 hour, n (%) |  |  |  |
| Pregnant women | 767 (96%) | 395 (99%) | 372 (93%) |
| Others | 32 (4%) | 5 (1%) | 27 (7%) |
| Someone in household smokes, n (%) |  |  |  |
| Yes | 253 (32%) | 136 (34%) | 117 (29%) |
| No | 546 (68%) | 264 (66%) | 282 (71%) |

**Table S3.** Summary of 24-hour personal exposures to PM_2.5_, BC, and CO among pregnant women using biomass at the HAPIN India IRC (based on valid samples only and removed the highest 1% of the exposure measurements)

|  |  | **N** | **India IRC** | **N** | **Villupuram** | **N** | **Nagapattinam** |
| --- | --- | --- | --- | --- | --- | --- | --- |
| PM_2.5_ exposure  (μg/m^3^) | Median (IQR) | 707 | 75.2 (81.1) | 337 | 97.5 (97.0) | 370 | 58.9 (58.9) |
|  | Mean [Range] |  | 104.2 [9.4, 647.5] |  | 130.4 [16.8, 647.5] |  | 80.4 [9.4, 472.5] |
| BC exposure  (μg/m^3^) | Median (IQR) | 692 | 9.4 (10.5) | 327 | 13.6 (12.4) | 365 | 6.7 (7.1) |
|  | Mean [Range] |  | 12.3 [0.6, 53.4] |  | 16.3 (0.7, 53.4) |  | 8.8 (0.6, 49.7) |
| CO exposure  (ppm) | Median (IQR) | 737 | 0.8 (1.8) | 369 | 1.1 (2.1) | 368 | 0.6 (1.2) |
|  | Mean [Range] |  | 1.5 [0, 13.6] |  | 1.9 (0, 13.6) |  | 1.1 [0, 11.4] |

**Table S4**. Crude and adjusted effects of personal PM_2.5_ on BP (N = 715). Results are presented in estimate and 95% confidence interval.

|  | **Crude Association** | | | | **Adjusted Association** | | | |
| --- | --- | --- | --- | --- | --- | --- | --- | --- |
|  | **Estimate** | **p-value** | **95% CI** | **AIC** | **Estimate** | **p-value** | **95% CI** | **AIC** |
| ***Systolic Blood Pressure*** | | | | | | | | |
| Linear | 0.0041 | 0.0746 | (-0.0004, 0.0087) | 5189 | 0.0023 | 0.3281 | (-0.0023, 0.0069) | 5177 |
| Log linear | 0.73 | 0.0811 | (-0.0904, 1.5505) | 5190 | 0.4411 | 0.2985 | (-0.3913, 1.2734) | 5177 |
| Categorical [Ref. Quartile 1] | |  |  |  |  |  |  |  |
| Quartile 2 | -0.1864 | 0.8467 | (-2.0787, 1.7058) | 5194 | -0.4036 | 0.6772 | (-2.306, 1.4989) | 5180 |
| Quartile 3 | 0.6369 | 0.5078 | (-1.25, 2.5238) |  | 0.3044 | 0.7535 | (-1.5983, 2.2071) |  |
| Quartile 4 | 1.3054 | 0.1748 | (-0.5815, 3.1923) |  | 0.7824 | 0.4201 | (-1.1217, 2.6865) |  |
| ***Diastolic Blood Pressure*** | | | | | | | | |
| Linear | 0.003 | 0.117 | (-0.0008, 0.0069) | 4936 | 0.0023 | 0.231 | (-0.0015, 0.006) | 4882 |
| Log linear | 0.6523 | 0.0625 | (-0.0342, 1.3389) | 4935 | 0.6528 | 0.0584 | (-0.0231, 1.3287) | 4879 |
| Categorical [Ref. Quartile 1] | |  |  |  |  |  |  |  |
| Quartile 2 | -0.7228 | 0.369 | (-2.3012, 0.8557) | 4934 | -0.5609 | 0.4748 | (-2.101, 0.9791) | 4878 |
| Quartile 3 | 0.9194 | 0.2518 | (-0.6545, 2.4934) |  | 1.032 | 0.1888 | (-0.5082, 2.5722) |  |
| Quartile 4 | 1.3403 | 0.095 | (-0.2337, 2.9143) |  | 1.5151 | 0.054 | (-0.0263, 3.0565) |  |

***Note:***

*All models adjusted for age, BMI, mother’s highest level of education, household wealth index, gestational age, and season.*

**Table S5**. Crude and adjusted effects of personal BC on BP (N = 699). Results are presented in estimate and 95% confidence interval.

|  | **Crude Association** | | | | **Adjusted Association** | | | |
| --- | --- | --- | --- | --- | --- | --- | --- | --- |
|  | **Estimate** | **p-value** | **95% CI** | **AIC** | **Estimate** | **p-value** | **95% CI** | **AIC** |
| ***Systolic Blood Pressure*** | | | | | | | | |
| Linear | 0.0445 | 0.1447 | (-0.0153, 0.1044) | 5078 | 0.0284 | 0.3559 | (-0.032, 0.0888) | 5066 |
| Log linear | 0.5332 | 0.1993 | (-0.2816, 1.348) | 5079 | 0.33 | 0.4392 | (-0.507, 1.167) | 5066 |
| Categorical [Ref. Quartile 1] | |  |  |  |  |  |  |  |
| Quartile 2 | 0.32 | 0.7431 | (-1.596, 2.236) | 5081 | 0.3304 | 0.7338 | (-1.5764, 2.2371) | 5068 |
| Quartile 3 | 1.0594 | 0.2787 | (-0.8594, 2.9782) |  | 1.0504 | 0.2849 | (-0.8766, 2.9775) |  |
| Quartile 4 | 1.5676 | 0.1087 | (-0.3484, 3.4837) |  | 1.1592 | 0.242 | (-0.7845, 3.1028) |  |
| ***Diastolic Blood Pressure*** | | | | | | | | |
| Linear | 0.0258 | 0.315 | (-0.0246, 0.0761) | 4837 | 0.0271 | 0.282 | (-0.0223, 0.0765) | 4785 |
| Log linear | 0.3861 | 0.2689 | (-0.299, 1.0712) | 4836 | 0.5038 | 0.1487 | (-0.1804, 1.1881) | 4784 |
| Categorical [Ref. Quartile 1] | |  |  |  |  |  |  |  |
| Quartile 2 | 0.0362 | 0.9648 | (-1.5747, 1.6471) | 4839 | 0.2138 | 0.7876 | (-1.344, 1.7717) | 4786 |
| Quartile 3 | 0.8531 | 0.2995 | (-0.7601, 2.4663) |  | 1.2862 | 0.1092 | (-0.2882, 2.8606) |  |
| Quartile 4 | 1.1181 | 0.1734 | (-0.4928, 2.729) |  | 1.2893 | 0.1114 | (-0.2987, 2.8773) |  |

***Note:***

*All models adjusted for age, BMI, mother’s highest level of education, household wealth index, gestational age, and season.*

**Table S6**. Crude and adjusted effects of personal CO on BP (N = 730). Results are presented in estimate and 95% confidence interval.

|  | **Crude Association** | | | | **Adjusted Association** | | | |
| --- | --- | --- | --- | --- | --- | --- | --- | --- |
|  | **Estimate** | **p-value** | **95% CI** | **AIC** | **Estimate** | **p-value** | **95% CI** | **AIC** |
| ***Systolic Blood Pressure*** | | | | | | | | |
| Linear | 0.1073 | 0.3007 | (-0.0961, 0.3108) | 5267 | 0.0874 | 0.3966 | (-0.115, 0.2898) | 5261 |
| Log linear | 0.4168 | 0.0454 | (0.0086, 0.8251) | 5265 | 0.3903 | 0.0604 | (-0.0171, 0.7976) | 5259 |
| Categorical [Ref. Quartile 1] | |  |  |  |  |  |  |  |
| Quartile 2 | 1.6966 | 0.0686 | (-0.13, 3.5233) | 5267 | 1.6371 | 0.0785 | (-0.1868, 3.461) | 5261 |
| Quartile 3 | 1.9329 | 0.0381 | (0.1063, 3.7595) |  | 1.9632 | 0.0342 | (0.1464, 3.78) |  |
| Quartile 4 | 1.4536 | 0.1182 | (-0.3706, 3.2777) |  | 1.1915 | 0.2019 | (-0.6398, 3.0228) |  |
| ***Diastolic Blood Pressure*** | | | | | | | | |
| Linear | 0.0795 | 0.3639 | (-0.0923, 0.2512) | 5020 | 0.0833 | 0.3292 | (-0.0842, 0.2507) | 4985 |
| Log linear | 0.3188 | 0.0699 | (-0.026, 0.6635) | 5018 | 0.3595 | 0.0365 | (0.0226, 0.6963) | 4981 |
| Categorical [Ref. Quartile 1] | |  |  |  |  |  |  |  |
| Quartile 2 | 1.0543 | 0.1795 | (-0.4862, 2.5948) | 5019 | 1.1015 | 0.1515 | (-0.4047, 2.6078) | 4981 |
| Quartile 3 | 1.351 | 0.0856 | (-0.1895, 2.8915) |  | 1.6005 | 0.0366 | (0.1002, 3.1009) |  |
| Quartile 4 | 1.9126 | 0.0149 | (0.3742, 3.451) |  | 2.0838 | 0.007 | (0.5714, 3.5961) |  |

***Note:***

*All models adjusted for age, BMI, mother’s highest level of education, household wealth index, gestational age, and season.*

**Table S7**. Full adjusted log-linear model output.

| **Coefficients:** | **Estimate** | **Std. Error** | **t value** | **Pr(>\|t\|)** |  |
| --- | --- | --- | --- | --- | --- |
| ***SBP - PM_2.5_*** | | | | | |
| (Intercept) | 101.44493 | 3.79531 | 26.729 | <2.00E-16 | *** |
| log(ECM_grav_neph_conc_M) | 0.44106 | 0.42393 | 1.04 | 0.298513 |  |
| MAyears_at_baseline | -0.07907 | 0.0953 | -0.83 | 0.406993 |  |
| m14_bmi | 0.43012 | 0.11207 | 3.838 | 0.000135 | *** |
| m10_educ_R2 | -0.9971 | 0.85687 | -1.164 | 0.244961 |  |
| m10_educ_R3 | -2.26222 | 0.83209 | -2.719 | 0.006715 | ** |
| factor(NationalQuintile)2 | -1.3074 | 0.85787 | -1.524 | 0.127955 |  |
| factor(NationalQuintile)3 | -1.47908 | 1.01684 | -1.455 | 0.146232 |  |
| factor(NationalQuintile)4 | -3.47045 | 1.69248 | -2.051 | 0.040685 | * |
| GAweeks_AT_baseline | -0.17077 | 0.11213 | -1.523 | 0.128201 |  |
| season1Summer | -2.0748 | 0.81381 | -2.549 | 0.010999 | * |
| season1Winter | -0.15274 | 1.07127 | -0.143 | 0.886663 |  |
| ***DBP - PM_2.5_*** | | | | | |
| (Intercept) | 54.1326 | 3.08217 | 17.563 | <2.00E-16 | *** |
| log(ECM_grav_neph_conc_M) | 0.65279 | 0.34428 | 1.896 | 0.0584 | . |
| MAyears_at_baseline | 0.09116 | 0.0774 | 1.178 | 0.2392 |  |
| m14_bmi | 0.56297 | 0.09101 | 6.185 | 1.05E-09 | *** |
| m10_educ_R2 | -0.44697 | 0.69587 | -0.642 | 0.5209 |  |
| m10_educ_R3 | -0.94751 | 0.67574 | -1.402 | 0.1613 |  |
| factor(NationalQuintile)2 | -0.9979 | 0.69667 | -1.432 | 0.1525 |  |
| factor(NationalQuintile)3 | -1.73759 | 0.82578 | -2.104 | 0.0357 | * |
| factor(NationalQuintile)4 | -1.76173 | 1.37446 | -1.282 | 0.2004 |  |
| GAweeks_AT_baseline | -0.4256 | 0.09106 | -4.674 | 3.54E-06 | *** |
| season1Summer | -2.59734 | 0.6609 | -3.93 | 9.33E-05 | *** |
| season1Winter | -0.79779 | 0.86998 | -0.917 | 0.3594 |  |
| ***SBP - BC*** | | | | | |
| (Intercept) | 103.53506 | 3.43978 | 30.099 | <2.00E-16 | *** |
| log(ECM_bc_conc_M) | 0.32998 | 0.4263 | 0.774 | 0.439158 |  |
| MAyears_at_baseline | -0.1066 | 0.09571 | -1.114 | 0.265774 |  |
| m14_bmi | 0.43165 | 0.11376 | 3.794 | 0.000161 | *** |
| m10_educ_R2 | -0.93865 | 0.88203 | -1.064 | 0.287616 |  |
| m10_educ_R3 | -2.20785 | 0.85055 | -2.596 | 0.009639 | ** |
| factor(NationalQuintile)2 | -1.26045 | 0.87102 | -1.447 | 0.148326 |  |
| factor(NationalQuintile)3 | -1.51273 | 1.03364 | -1.463 | 0.143791 |  |
| factor(NationalQuintile)4 | -3.40507 | 1.70074 | -2.002 | 0.045665 | * |
| GAweeks_AT_baseline | -0.19362 | 0.11371 | -1.703 | 0.089065 | . |
| season1Summer | -2.14931 | 0.82463 | -2.606 | 0.009349 | ** |
| season1Winter | 0.10126 | 1.08926 | 0.093 | 0.925962 |  |
| ***DBP - BC*** | | | | | |
| (Intercept) | 55.79791 | 2.81222 | 19.841 | <2.00E-16 | *** |
| log(ECM_bc_conc_M) | 0.50384 | 0.34852 | 1.446 | 0.149 |  |
| MAyears_at_baseline | 0.07782 | 0.07825 | 0.994 | 0.32 |  |
| m14_bmi | 0.5703 | 0.09301 | 6.132 | 1.47E-09 | *** |
| m10_educ_R2 | -0.45632 | 0.72111 | -0.633 | 0.527 |  |
| m10_educ_R3 | -0.94859 | 0.69537 | -1.364 | 0.173 |  |
| factor(NationalQuintile)2 | -0.9518 | 0.71211 | -1.337 | 0.182 |  |
| factor(NationalQuintile)3 | -1.72134 | 0.84506 | -2.037 | 0.042 | * |
| factor(NationalQuintile)4 | -1.74637 | 1.39045 | -1.256 | 0.21 |  |
| GAweeks_AT_baseline | -0.41239 | 0.09296 | -4.436 | 1.07E-05 | *** |
| season1Summer | -2.65064 | 0.67419 | -3.932 | 9.29E-05 | *** |
| season1Winter | -0.84955 | 0.89053 | -0.954 | 0.34 |  |
| ***SBP - CO*** | | | | | |
| (Intercept) | 106.59989 | 3.04755 | 34.979 | <2.00E-16 | *** |
| log(CO_avg_ppm_M) | 0.39025 | 0.20747 | 1.881 | 0.0604 | . |
| MAyears_at_baseline | -0.07845 | 0.09086 | -0.863 | 0.3882 |  |
| m14_bmi | 0.25638 | 0.10947 | 2.342 | 0.0195 | * |
| m10_educ_R2 | -1.18672 | 0.81861 | -1.45 | 0.1476 |  |
| m10_educ_R3 | -1.94773 | 0.80629 | -2.416 | 0.016 | * |
| factor(NationalQuintile)2 | -0.75009 | 0.83261 | -0.901 | 0.3679 |  |
| factor(NationalQuintile)3 | -1.69813 | 1.00477 | -1.69 | 0.0915 | . |
| factor(NationalQuintile)4 | -2.8689 | 1.66619 | -1.722 | 0.0855 | . |
| GAweeks_AT_baseline | -0.19371 | 0.10938 | -1.771 | 0.077 | . |
| season1Summer | -1.59634 | 0.78325 | -2.038 | 0.0419 | * |
| season1Winter | 1.39836 | 1.07741 | 1.298 | 0.1947 |  |
| ***DBP - CO*** | | | | | |
| (Intercept) | 59.11512 | 2.52036 | 23.455 | <2.00E-16 | *** |
| log(CO_avg_ppm_M) | 0.35947 | 0.17158 | 2.095 | 0.0365 | * |
| MAyears_at_baseline | 0.06541 | 0.07514 | 0.87 | 0.3844 |  |
| m14_bmi | 0.4483 | 0.09054 | 4.952 | 9.18E-07 | *** |
| m10_educ_R2 | -0.30215 | 0.677 | -0.446 | 0.6555 |  |
| m10_educ_R3 | -0.96536 | 0.66681 | -1.448 | 0.1481 |  |
| factor(NationalQuintile)2 | -0.39402 | 0.68858 | -0.572 | 0.5674 |  |
| factor(NationalQuintile)3 | -1.29979 | 0.83096 | -1.564 | 0.1182 |  |
| factor(NationalQuintile)4 | -1.05257 | 1.37797 | -0.764 | 0.4452 |  |
| GAweeks_AT_baseline | -0.41561 | 0.09046 | -4.594 | 5.13E-06 | *** |
| season1Summer | -2.09114 | 0.64776 | -3.228 | 0.0013 | ** |
| season1Winter | 0.19468 | 0.89104 | 0.218 | 0.8271 |  |

***Note:***

*^a.^ Study site: Nagapattinam is the reference group; ^b.^ Mother’s highest level of education: No formal education or Primary school incomplete is the reference group (1), Primary school complete or Secondary school incomplete (2), Secondary school complete or Vocational or Some college or university (3); ^c.^ Household wealth at national quintiles: Lowest (1) is the reference group; ^d.^ Gestational age in weeks at baseline; ^e.^ Season of the baseline exposure and blood pressure measurement: Monsoon is the reference group.*

*Significance codes: ‘***’ 0.001 ‘**’ 0.01 ‘*’ 0.05 ‘.’ 0.1*

**Table S8.** Sensitivity analysis results of adjusted generalized additive models: approximate significance of exposure smooth terms.

|  | SBP | | | | DBP | | | |
| --- | --- | --- | --- | --- | --- | --- | --- | --- |
|  | edf | F | p-value | AIC | edf | F | p-value | AIC |
| PM_2.5_ | 1.001 | 0.954 | 0.329 | 5177 | 1.344 | 0.738 | 0.355 | 4882 |
| BC | 1.000 | 0.852 | 0.356 | 5066 | 1.967 | 1.521 | 0.208 | 4784 |
| CO | 1.006 | 0.704 | 0.400 | 5261 | 1.789 | 0.985 | 0.410 | 4985 |

***Note:*** *Presented results based on GAMs using thin plate regression splines with 4 degrees of freedom.*

**Table S9.** Adjusted association between personal PM_2.5_ (N = 707), BC (N = 692) and CO (N = 722) and BP. Results are presented in estimate and 95% confidence interval.

|  |  | **Estimate** | **p-value** | **95% CI** | **AIC** |
| --- | --- | --- | --- | --- | --- |
| ***Systolic Blood Pressure*** | | | | | |
| PM_2.5_ | Log linear | 0.3483 | 0.4387 | (-0.5343, 1.2308) | 5121 |
|  | Exposure Quartiles [Ref. Quartile 1] | | |  |  |
|  | Quartile 2 | -0.4237 | 0.6625 | (-2.3288, 1.4814) | 5124 |
|  | Quartile 3 | 0.2849 | 0.7691 | (-1.6204, 2.1903) |  |
|  | Quartile 4 | 0.6699 | 0.4946 | (-1.2548, 2.5946) |  |
| BC | loglinear | 0.376 | 0.3954 | (-0.4922, 1.2442) | 5020 |
|  | Exposure Quartiles [Ref. Quartile 1] | | |  |  |
|  | Quartile 2 | 0.3324 | 0.7333 | (-1.5819, 2.2466) | 5023 |
|  | Quartile 3 | 1.0606 | 0.2821 | (-0.8741, 2.9954) |  |
|  | Quartile 4 | 1.2218 | 0.2244 | (-0.751, 3.1946) |  |
| CO | loglinear | 0.3488 | 0.1018 | (-0.0692, 0.7669) | 5200 |
|  | Exposure Quartiles [Ref. Quartile 1] | | |  |  |
|  | Quartile 2 | 1.6278 | 0.0798 | (-0.1938, 3.4494) | 5201 |
|  | Quartile 3 | 1.9517 | 0.0351 | (0.1372, 3.7662) |  |
|  | Quartile 4 | 0.9969 | 0.2903 | (-0.8524, 2.8463) |  |
| ***Diastolic Blood Pressure*** | | | | | |
| PM_2.5_ | loglinear | 0.6265 | 0.0867 | (-0.0905, 1.3436) | 4828 |
|  | Exposure Quartiles [Ref. Quartile 1] | | |  |  |
|  | Quartile 2 | -0.5706 | 0.468 | (-2.1135, 0.9723) | 4826 |
|  | Quartile 3 | 1.0239 | 0.1931 | (-0.5192, 2.567) |  |
|  | Quartile 4 | 1.4574 | 0.0668 | (-0.1014, 3.0162) |  |
| BC | loglinear | 0.7056 | 0.0501 | (-0.0004, 1.4117) | 4734 |
|  | Exposure Quartiles [Ref. Quartile 1] | | |  |  |
|  | Quartile 2 | 0.2247 | 0.7769 | (-1.3318, 1.7813) | 4737 |
|  | Quartile 3 | 1.2999 | 0.1052 | (-0.2733, 2.8731) |  |
|  | Quartile 4 | 1.5498 | 0.0583 | (-0.0543, 3.1539) |  |
| CO | loglinear | 0.3617 | 0.0411 | (0.0147, 0.7086) | 4931 |
|  | Exposure Quartiles [Ref. Quartile 1] | | |  |  |
|  | Quartile 2 | 1.1013 | 0.1528 | (-0.4095, 2.612) | 4931 |
|  | Quartile 3 | 1.5998 | 0.0372 | (0.0949, 3.1046) |  |
|  | Quartile 4 | 2.0752 | 0.0081 | (0.5414, 3.609) |  |

***Note:***

*All models adjusted for age, BMI, mother’s highest level of education, household wealth index, gestational age, and season.*
